# Elevated CO_2_ and temperature under future climate change increase severity of rice sheath blight

**DOI:** 10.3389/fpls.2023.1115614

**Published:** 2023-01-26

**Authors:** Min Shen, Chuang Cai, Lian Song, Jiangbo Qiu, Chuanqi Ma, Dongming Wang, Xinyue Gu, Xiong Yang, Wei Wei, Ye Tao, Jishuang Zhang, Gang Liu, Chunwu Zhu

**Affiliations:** ^1^ State Key Laboratory of Soil and Sustainable Agriculture, Institute of Soil Science, Chinese Academy of Sciences, Nanjing, China; ^2^ University of Chinese Academy of Sciences, Beijing, China; ^3^ School of Environmental and Civil Engineering, Jiangnan University, Wuxi, China

**Keywords:** *Oryza sativa* L., sheath blight, *Rhizoctonia solani*, free-air CO^2^ enrichment, elevated temperature, T-FACE, yield

## Abstract

Sheath blight (ShB), caused by *Rhizoctonia solani*, is one of the major threats to rice (*Oryza sativa* L.) production. However, it is not clear how the risk of rice ShB will respond to elevated CO_2_ and temperature under future climate change. Here, we conducted, field experiments of inoculated *R. solani* under combinations of two CO_2_ levels (ambient and enriched up to 590 μmol mol^-1^) and two temperature levels (ambient and increased by 2.0°C) in temperature by free-air CO_2_ enrichment (T-FACE) system for two cultivars (a susceptible cultivar, Lemont and a resistant cultivar, YSBR1). Results indicate that for the inoculation of plants with *R. solani*, the vertical length of ShB lesions for cv. Lemont was significantly longer than that for cv. YSBR1 under four CO_2_ and temperature treatments. The vertical length of ShB lesions was significantly increased by elevated temperature, but not by elevated CO_2_, for both cultivars. The vertical length of ShB lesions under the combination of elevated CO_2_ and elevated temperature was increased by 21–38% for cv. Lemont and by -1–6% for cv. YSBR1. A significant increase in MDA level was related to a significant increase in the vertical length of ShB lesions under the combination of elevated CO_2_ and elevated temperature. Elevated CO_2_ could not compensate for the negative effect of elevated temperature on yield of both cultivars under future climate change. Rice yield and biomass were further decreased by 2.0–2.5% and 2.9–4.2% by an increase in the severity of ShB under the combination of elevated CO_2_ and elevated temperature. Thus, reasonable agronomic management practices are required to improve both resistance to ShB disease and grain yield for rice under future climate change.

## 1 Introduction

Rice (*Oryza sativa* L.), one of the most important food crops, serves as the staple food for approximately 67% of the world population (Senapati et al., 2022). Sheath blight (ShB), caused by *Rhizoctonia solani* Kühn (*Thanatephorus cucumeris* (Frank) Donk), is a potentially devastating rice disease in all temperate and tropical rice production regions, especially in irrigated production systems (Gangopadhyay and Chakrabarti, 1982; Lee and Rush, 1983; Ou, 1985; Dath, 1990; Rush and Lee, 1992; Rodrigues et al., 2001). The disease was first reported in Japan in 1910 and has subsequently spread to almost all rice-growing area in the world (Rush and Lee, 1992; Senapati et al., 2022). Economic losses in rice yields up to 58% have reportedly been demonstrated, due to widespread occurrence (Chahal et al., 2003).

Meteorological factors, such as air temperature, could directly influence the growth of *R. solani* in the field (Castilla et al., 1996; Thind et al., 2008; Biswas et al., 2011; Bhukal et al., 2015; Senapati et al., 2022). Correlation analysis showed that high air temperature associated with high relative humidity in the forenoon is a major predisposing factor for the development of ShB in rice (Castilla et al., 1996; Biswas et al., 2011). Maximum spread of ShB was observed in a temperature range of 25–30°C and relative humidity of 80–100%, and a maximum temperature around 34°C and a minimum temperature around 26°C were found to be favorable for the dispersal of ShB after its establishment in the field (Thind et al., 2008; Biswas et al., 2011; Bhukal et al., 2015; Senapati et al., 2022). Among the meteorological factors, atmospheric CO_2_ concentration and air temperature have been increasing rapidly, and probably the CO_2_ level will reach 500–1000 μmol mol^-1^ and the air temperature will increase by 1.0–3.7°C by the end of this century (Ciais et al., 2013). Increased air temperatures are expected to have a significant impact on plant diseases (Chakraborty et al., 2000; Chakraborty, 2001). Manning and Tiedermann (1995) pointed out that elevated CO_2_ increased plant size and canopy density, and thus the canopy microclimate favored the development of various diseases. Elevated CO_2_ had no effect on the incidence of the panicle blast phase of the disease, but increased the incidence of naturally sheath blight in rice (Kobayashi et al., 2006). Meanwhile, Kobayashi et al. (2006) suggested that the impacts of global increase in atmospheric CO_2_ on crop production in the future must take into account in the increased risks of the plant to diseases. However, it is still unclear whether elevated CO_2_ and temperature will increase the severity of rice ShB under future climate conditions and thus lead to rice yield losses.

The T- FACE (temperature by free- air CO_2_ enrichment) system can best mimic the elevated CO_2_ and temperature conditions of future climates and provides a unique opportunity to evaluate the “real” effects of elevated CO_2_ and/or temperature on rice ShB risk, as this system allows crops and *R. solani* to be grown under elevated CO_2_ and temperature condition in natural and fully open fields. Kobayashi et al. (2006) reported that FACE (free-air CO_2_ enrichment) system used in the field without any enclosures, could minimize the artificial effect that is inevitable with the enclosure approach. Thompson and Drake (1994) found that the severity of rust in *Scirpus olneyi* plants may have been lower in the enclosed chambers, such as growth chambers and cabinets, than in the field plots, considering the barrier that the enclosed chambers present. In addition, enclosures alter microclimate for the plants and may change the responses of plant to elevated CO_2_ (McLeod and Long, 1999). The effects of elevated CO_2_ and temperature on crop growth response also differed greatly between studies in enclosed chambers and T-FACE (Long et al., 2004; Long et al., 2006), which may result in different responses of rice ShB to elevated CO_2_ and temperature. This is because growth of *R. solani* needs certain carbon and nitrogen sources provided by rice plants (Chen et al., 2002; Wang et al., 2004). However, the responses of rice ShB to elevated CO_2_ and temperature have not yet been quantified in T-FACE systems.

Susceptibility of rice to *R. solani* can be affected by rice morphological parameters, such as plant height, stem thickness and tiller angle, length and width of flag leaf, days to heading and planting density (Senapati et al., 2022). Some studies found that the susceptibility of rice to *R. solani* was also related to rice physiological parameters, such as carbohydrate metabolism, phenolic compounds, flavonoids, lignin, membrane lipid peroxidation (MDA) level and POD, PPO and SOD activities (Ertani et al., 2011; Zuo et al., 2014a; Zuo et al., 2014b; Molla et al., 2020). Previous studies found that elevated CO_2_ increased the number of tillers per plant, which increased the likelihood of ShB disease spread between more densely packed neighbouring plants (Kobayashi et al., 2006). Moreover, elevated CO_2_ was reported to reduce leaf nitrogen content, but increase the C:N ratio, total phenolics and tannins, which probably reduced disease severity (Eastburn et al., 2011). In addition, elevated temperature accelerated plant growth and developmental rates, which modified canopy architecture and affected pathogen development (Pangga et al., 2013).

The main objectives of this study are: (i) to analyze whether elevated CO_2_ and temperature increase ShB disease severity in rice, (ii) if so, which morphological and physiological parameters are associated with the increase in ShB disease severity, and (iii) to what extent growth and yield losses are caused by the increase in ShB disease severity. To this end, ShB lesion length, morphological and physiological parameters, above-ground biomass, and grain yields were therefore assessed for two rice cultivars, cvs Lemont and YSBR1 over two growing seasons under T- FACE environments. Of the two cultivars, cv. Lemont is a ShB disease susceptible variety, while cv. YSBR1 is a ShB disease resistant variety. Therefore, we also aim to investigate whether there is any difference between the two cultivars in terms of the effects of elevated CO_2_ and temperature on severity of ShB disease. Such information would provide the basis for selecting rice cultivars to reduce yield losses caused by ShB disease and to ensure food security under future climates with elevated CO_2_ and temperature.

## 2 Materials and methods

### 2.1 Experiment site and detailed conditions of the T-FACE system

**Figure 1:**
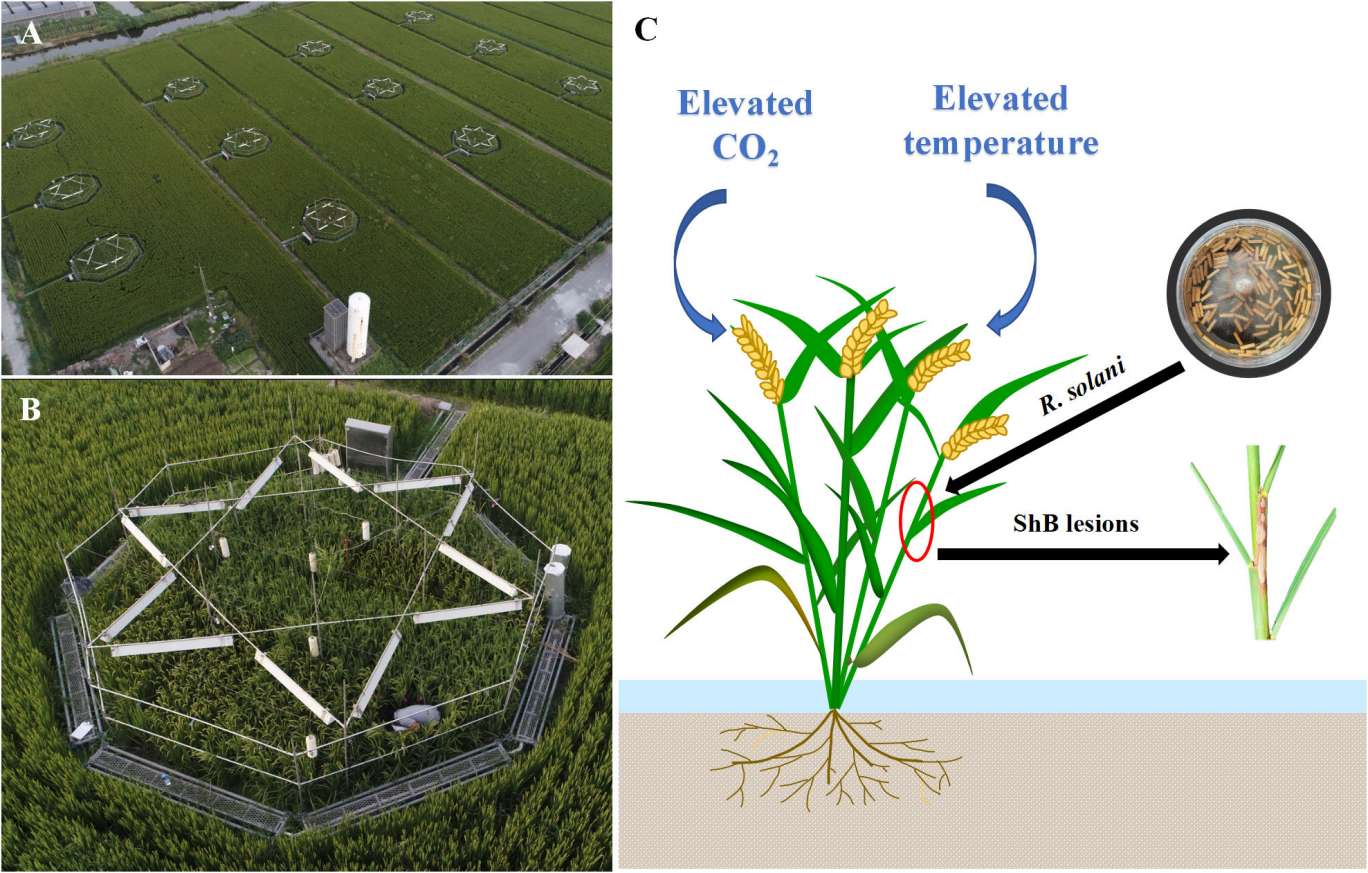
**(A, B)** In this T-FACE experiment, four treatments were designed with twelve octagonal plots: two CO_2_ levels (ambient and enriched up to 590 μmol mol^-1^) and two temperature levels (ambient and increased by 2.0°C). Each treatment was set three repetitions. **(C)** For inoculation experiment, the inoculum with *R. solani* was placed 1 cm depth inside the third sheath from top, then plant was infected forming ShB lesions.

Field experiments were conducted in the T-FACE system, established in Kangbo village (31°30’N, 120°33’E), Guli Township, Changshu Municipality, Jiangsu, China. The area is located in the centre of the Tai Lake Plain, and has a subtropical monsoon climate. The T-FACE system had twelve octagonal plots located in different sites with similar soils and agronomic history (Liu et al., 2014; Zhang et al., 2015; Cai et al., 2016) (**Figures 1A, B**). More detailed information of the T-FACE system was described by Cai et al. (2016); Cai et al., 2018; Cai et al. (2019). Four treatments designed, one with targeted atmospheric CO_2_ up to 590 μmol mol^-1^ during daytime (C+T), one with canopy temperature warming 2.0°C above ambient (CT+), and one with combined CO_2_ enrichment and warming (C+T+), with an untreated plot with ambient condition as control (CT). Each treatment was set three replications (**Figures 1A, B**). Daily data for air temperature (sensor: HMP155A; Campbell Scientific Inc., Logan, UT, USA) and global solar radiation (sensor: LI190SB; Li-COR Inc., Lin-coln, NE, USA) under ambient condition were recorded by the weather station at the experimental site, using the CR1000 Campbell datalogger (Campbell Scientific Inc.). Information of the daytime and nighttime increases in canopy temperature implemented under CT+ and C+T+, and the daytime increase in CO_2_ concentration implemented under C+T and C+T+ were also record by using the CR1000 Campbell datalogger (**Table 1**). Meanwhile, information on weather conditions such as mean daily air temperature and global solar radiation under ambient conditions, and level of nitrogen (N) application over the entire rice growing cycle under four CO_2_ and temperature treatments are summarized in **Table 1**.

**Table 1:** Summary of environment conditions in T-FACE experiments of 2020 and 2021^a^.

	2020	2021
Daytime CO_2_ concentration increment relative to CT (μmol mol^-1^)		
C+T	190 (32)	190 (38)
C+T+	205 (39)	183 (28)
Canopy temperature increment relative to CT (°C)		
CT+	1.7 (1.1)	2.2 (1.0)
C+T+	1.6 (0.7)	1.8 (0.5)
Mean daily air temperature for CT (°C)	25.1 (4.0)	26.7 (2.4)
Mean daily global radiation (MJ m^-2^ day^-1^)	13.1 (6.1)	13.6 (6.3)
Basal N		
Time of N application (day)^b^	0	-3
Amount of N application (g m^-2^)	6.9	6.9
Top-dressed N^c^		
Time of N application (day)^b^	12; 48	33; 53
Amount of N application (g m^-2^)	6.0; 5.2	6.0; 5.2

a CT, C+T, CT+ and C+T+ stand for ambient condition, elevated CO_2_, elevated temperature and the combination of elevated CO_2_ and elevated temperature, respectively. Weather data in the table represent seasonal average values (SD between the daily values in brackets).

b The time of N application is expressed in days after transplanting.

c Top‐dressed N was applied before the stem‐elongating stage in rice, and was split into two applications (indicated by the pair of data therein in the table).

### 2.2 Crop cultivation

In the 2020 and 2021 rice experimental seasons, two rice cultivars cvs Lemont (a *japonica* subspecies), and YSBR1 (an *indica* subspecies), were grown in field. Cv. Lemont is susceptible to rice ShB, while cv. YSBR1 shows high resistance to rice ShB (Zuo et al., 2009; Xue et al., 2016). For cvs Lemont and YSBR1, seeds were first germinated in the field under the same ambient condition. And then three-leaf-stage seedings were manually transplanted at a density of two seedings per hill for both two cultivars on June 22 (2020) and June 26 (2021). The spacing between hills was set at 16.7 cm × 25 cm for both two cultivars (equivalent to 24 hills m^-2^).

### 2.3 Pathogen inoculation and lesion survey

For ShB fungus inoculation, the *YN*-*7* isolate, *R. solani* with moderate pathogenicity was used in this study and several previous studies (Zuo et al., 2008; Zhu et al., 2014; Chen et al., 2016; Xue et al., 2016). Briefly, autoclaved, truncated, thin matchsticks with a length of 1.0 cm and a width of 2 mm were incubated with *YN*-*7* on potato dextrose broth (PDB) medium for 3 to 4 days in the dark at 28°C, based on Zou et al. (2000). Secondly, the woody matchsticks colonized by the ShB fungus were used as the inoculum, according to Xue et al. (2016). Then, the inoculation in the field was conducted at the booting stage of rice (Wang et al., 2011; Zuo et al., 2013). Each treatment was planted in three plots (replicates) and 48 hills in 2 m^2^ were planted in each plot for both cultivars cvs Lemont and YSBR1. Half of each plot under four CO_2_ and temperature treatments was used for inoculation. In each subplot for inoculation, two rice cultivars were planted in six rows, with 8 hills/row, and the central 12 hills for each cultivar were inoculated with the inoculum of *R. solani*. Inoculum of *R*. solani was placed 1 cm depth inside the third sheath, counting from top to bottom without changing the sheath-stem holding status for three main stems per hill, according to Zuo et al. (2013) (**Figure 1C**). In both 2020 and 2021 T-FACE experiments, inoculation of *R. solani* was performed on August 17 (at the booting stage). Disease severity was represented by the vertical length of ShB lesions referring to the total length of lesions with inoculated stem in the vertical direction (Zuo et al., 2009). The lesion length was measured with a ruler on August 21, 23 and September 4, 2020, and August 21, 23, 25 and September 4, 2021.

### 2.4 Plant sampling and data collection

#### 2.4.1 Measurement of morphological parameters

For plants of both cultivars in uninoculated area of each subplot, we measured their morphological parameters including tiller number (TN), plant height (PH), flag leaf length (FLL), flag leaf width (FLW), stem diameter (SSD), stem wall thickness (SWT) and specific leaf weight (SLW) on Aug. 26, 2021. For each cultivar, we counted tiller number for 12 hills in each subplot. Then PH was measured from the soil surface to the tips of the tallest flag leaves of each hill with five replicates in each subplot. FLL and FLW were measured as the length and widest length with five flag leaves as replicates in each subplot. SSD and SWT were measured with cross type by using vernier caliper with five main stems as replicates in each subplot. SLW was calculated as the ratio of dry weight to flag leaf area with five flag leaves as replications in each subplot.

Then, three representative hills of each subplot were selected and destructively sampled on August 26, 2021. The samples were separated into leaves and stems (including leaf sheaths). Three fresh leaf sheaths from each subplot were stored at -20 °C and then plant parts were oven-dried (DHG-9240A, China) at 105°C for 30 minutes and then at 80°C until constant weight. Firstly, the wax content of sheath (WCS) was measured by cutting the fresh leaf sheath into pieces, placing 2.0 g (W) in a beaker, adding 30 ml of chloroform, and soaking for 1 minute. Finally, the soaking solution was filtered to the weighed beaker (W_1_) into the fume hood until the chloroform volatilization was completed, and the constant beaker (W_2_) was weighed again. The following formula was used to calculate the wax content of sheath:


(1) $$\text{\% Wax content = }\left({\text{W}}_{2–}{\text{W}}_{1}\right)\text{/W}$$

where W is the weight of sample, W_1_ is the weighed beaker mass (g), and W_2_ is the mass of beaker after chloroform volatilization (g) (Zhou et al., 2007; Zuo et al., 2014b).

In addition, a dry sample (0.5g) of fine powder was placed in a beaker, 12 ml of HNO_3_ and 3 ml HClO_4_ were added into each beaker including three empty ones (control), and then soaked overnight. Next day, all beakers were placed on a heating plate and the digestion temperature was set at 220 °C to digest the fine powder. When the solution layer was boiled to be clarified, the temperature was set to 300 °C to remove the excess acid. The cooling remaining solution was transferred into a 50 ml volumetric bottle and pure deionized water was added up to 50 ml. Finally, the Si content of leaf and stem was determined by High Performance Liquid Chromatography Inductively Couple Plasma Mass Spectrometry (HPLC-ICP-MS, 7700X, Agilent, Palo Alto, California, USA).

#### 2.4.2 Determination of physiological parameters

Physiological parameters, including carbohydrate and phenolic metabolites, membrane lipid peroxidation and antioxidant enzymes of the stems, were determined for inoculated and uninoculated areas under four CO_2_ and temperature treatments in 2021 T-FACE experiment. As the infection of *R. solani* formed the lesions on the stems and rapid development of lesion length occurred at the early stage of inoculation, we collected the fresh samples of inoculated and uninoculated stems (including leaf sheaths) and stored at -80°C on August 26 (10 days after inoculation) for determinations of these physiological parameters.

##### 2.4.2.1 Carbohydrate metabolites

Soluble sugar, starch and cellulose contents of stems were determined. Some carbohydrates can provide some carbon nutrition for *R. solani* (Wang et al., 2004). Firstly, the soluble sugar content of fresh stems was determined using the anthrone colorimetry method as described by Fairbairn (1953). Essentially, 0.2 g of fresh stem was cut into pieces and placed in a tube with 10 ml pure water, then the solution was boiled twice for 30 minutes. The solution was filtered and washed several times and fixed into a 50 ml volumetric flask. The next, 0.5 ml solution was taken and 1.5 ml pure water was added for determination, 0.5 ml anthrone and 5 ml sulphuric acid were added and mixed well. Finally, the solution was subjected to color-reaction at 100°C for 1 minute, and then was placed at room temperature for 10 –15 minutes to cool down, and the optical density was measured at 620 nm by using UV-Visible Spectrophotometer (Thermo Evolution 201, Waltham, MA, USA). The content of soluble sugar was expressed as percentage of fresh weight (% FW).

Secondly, the starch of stems was determined by acid hydrolysis method using the kit (G0507W, Suzhou Grace Bio-technology Co., LTD, Suzhou, China) (Luo et al., 2020). The frozen samples of stems were grounded to powder in liquid nitrogen, and 0.1 g sample was added to 1 mL of 80% ethanol and extracted at 80 °C water bath for 30 minutes, the extraction buffer was centrifuged at 3000 rpm for 5 minutes at room temperature. The extraction was decomposed into glucose, and then the glucose content was determined by anthrone colorimetry. Then the determination wavelength is 620 nm by UV-Visible Spectrophotometer (Thermo Evolution 201, Waltham, MA, USA). The content of starch was expressed as percentage of fresh weight (% FW).

Finally, the cellulose content of stems was determined using the kit (G0715W, Suzhou Grace Bio-technology Co. LTD, Suzhou, China) based on hydrolysis and dehydration of cellulose into furfural compounds, which can react with anthrone (Fan et al., 2021). Briefly, 0.1 g of fresh sample was weighed, and 1.5 mL of 80% ethanol was added to grind the homogenate. The extraction was taken at 50 °C in water bath for 20 minutes and took out the running water and cooled it. Then the extraction buffer was centrifuged at 12000 rpm at 25 °C for 10 minutes and discarded the supernatant to keep the sediment. The sediment was added 1mL acetone to centrifuge at 12000 rpm at 25 °C for 10 minutes and the supernatant was discarded to keep the sediment. Then the starch was removed from sediment and acid buffer was added. The mixed solution was centrifuged at 8000 rpm at 25 °C for 5 minutes, and the anthrone reagent was added to the supernatant. The optical density of solution was measured at 620 nm by using UV-Visible Spectrophotometer (Thermo Evolution 201, Waltham, MA, USA). The content of cellulose was expressed as percentage of fresh weight (% FW).

##### 2.4.2.2 Phenolic metabolites

Total phenol, flavonoid and lignin contents of the stems were determined. Firstly, total phenol content of stems was determined colorimetrically using the Folin-Ciocalteau’s method (Aires et al., 2011; Goufo et al., 2014), with modifications. The 60% methanol extract was mixed with FC reagent and developed with 7.5% Na_2_CO_3_. The mixture was shaken vigorously, and then heated at 45 °C for 15 minutes. The absorption value was measured at 760 nm by using a UV-Visible Spectrophotometer (Thermo Evolution 201, Waltham, MA, USA), to obtain the total phenol content of each sample. The content of total phenol was expressed in microgram gallic acid equivalents (GAE) per gram of fresh weight (μg g^-1^ FW).

Secondly, the flavonoid content of the stems is determined using a colorimetric method (Aires et al., 2011; Goufo et al., 2014), i.e., in alkaline nitrite solution, flavonoids and aluminum ions form a red complex with a characteristic absorption peak at 510 nm. The sample was weighed in 0.1 g of fresh stem, and ground and homogenized by adding 1.5 ml of 60% ethanol. Then the extraction buffer was shake at 60 °C for 2 hours and centrifuged at 12000 rpm at 25 °C for 10 minutes. Each extract was determined using 5% NaNO_2_ and 10% AlCl_3_. Then 1 mol L^-1^ NaOH and water were sequentially added. The reaction solution was vortex-mixed and the absorbance measured immediately at 510 nm with a spectrophotometer (Thermo Evolution 201, Waltham, MA, USA). Measurements were calibrated using a standard curve of prepared catechin solution, and the flavonoid content was expressed as mg of catechin equivalents per gram of fresh weight of stem (mg g^-1^ FW).

Thirdly, the lignin content of stems was determined using a kit (G0708W, Suzhou Grace Bio-technology Co. LTD, Suzhou, China) according to the manufacturer’s instructions, based on acetylation of lignin phenolic hydroxyl group (Fan et al., 2021). Briefly, an appropriate amount of the tissue sample was taken for drying and grinding, and passed it through 40 mesh sieves. Then, 1.5 mg of sieved powder tissue was mixed with 1.5 ml of 80% ethanol, and mixed well by vortex shaking. The solution was placed in a 50 °C water bath for 20 minutes, and the supernatant was taken out and cooled down, 12000 rpm and 25 °C for 10 minutes. The supernatant was discarded and the sediment was retained. The sediment was dried at 95 °C for standby. The UV-Visible Spectrophotometer (Thermo Evolution 201, Waltham, MA, USA) was preheated for 30 minutes by setting the temperature to 25 °C, and set the wavelength at 280 nm. The absorbance value at 280 nm is positively related to the lignin content. The content of lignin was expressed as percentage of dry weight (% DW).

##### 2.4.2.3 Membrane lipid peroxidation and antioxidant enzymes

Malondialdehyde (MDA) level, antioxidant enzymes including superoxide dismutase (SOD), peroxidase (POD), and polyphenol oxidase (PPO) activities in fresh stems was determined, the method of plant enzyme-linked immunosorbent was employed. Firstly, the concentration of protein in the stems was determined by bicinchoninic acid method using the BCA assay method (Beyotime Biotechnology, Haimen, China). Under alkaline conditions, the protein reduced Cu^2+^ to Cu^+^. Cu^+^ and bicinchoninic acid reagent formed a purple complex. The concentration of the protein can be calculated by the absorbance at 562 nm by spectrophotometer.

The next 0.5 g of fresh plant tissue was homogenized with 4.5 ml phosphate buffer (0.1 mol L^-1^, pH7.4) at 4°C. Then the crude extract was centrifuged at 3000 rpm for 20 minutes at 4°C and the supernatant was collected to measure MDA level, SOD, POD and PPO enzyme activities of stems by corresponding plant enzyme-linked immunosorbent assay (ELISA) kits (JL22715, JL22719, JL22742, and JL22725, Jianglai Biotechnology, Shanghai, China), respectively, according to the manufacturer’s instructions. Briefly, 40 μl of sample diluent was added to the sample hole to be measured on the enzyme coated plate and 10 μl of samples was added to be tested. 100 μl of enzyme labeled reagent was added to each hole. The plate was sealed with a sealing film and then incubated at 37 °C for 60 minutes. Each hole was filled with detergent and then discarded. Color developing agents were added into each hole in turn, shaken gently and mixed well, and put in the dark at 37 °C for 15 minutes. 50 μl of termination liquid was added to each hole. Finally, the absorbance of each hole was measured in sequence at 450 nm by using microplate reader (Multiskan Sky, Thermo, Waltham, MA, USA). The MDA level was expressed by as nanomole per gram protein (nmol mg^-1^ pro) and the SOD, POD and PPO activities were expressed as active unit per gram protein (U mg^-1^ pro).

### 2.5 Grain yield and above-ground biomass of rice

At maturity, four representative hills of both cvs Lemont and YSBR1 were harvested from inoculated and uninoculated areas in each plot, and then the grain yields and above-ground biomass of plants were determined.

### 2.6 Statistical analysis

Statistical analysis of the data was performed with SPSS 26 (IBM, USA), and the Pearson correlation test and graphs were generated by Origin 2022 (OriginLab Corporation, USA). Three-way analysis of variance (ANOVA) was used to examine the individual and combined effects of year, CO_2_ and temperature on the vertical length of rice ShB lesions, and the individual and combined effects of variety, CO_2_ and temperature on physiological parameters. Four-way analysis of variance (ANOVA) was used to examine the individual and combined effects of variety, inoculation, CO_2_ and temperature on physiological parameters. Mean comparisons (least significant difference) were used to reveal the difference in data between CO_2_ and temperature treatments. All results reported as significant had a *P<* 0.05.

## 3 Results

### 3.1 Vertical length of rice ShB lesions

**Figure 2:**
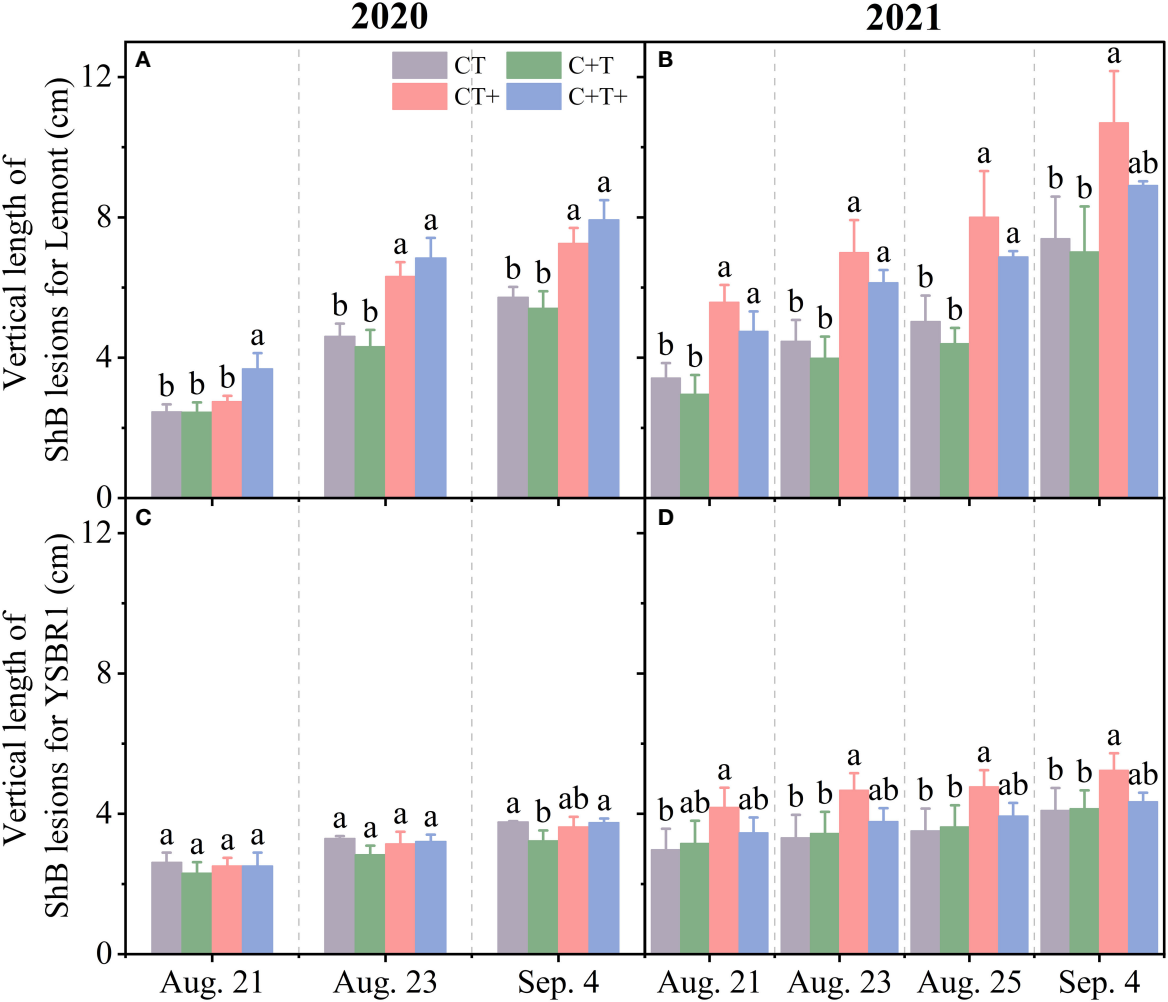
The vertical length of ShB lesions for inoculated plants of cvs Lemont **(A, B)** and YSBR1 **(C, D)** under ambient condition (CT), elevated CO_2_ (C+T), elevated temperature (CT+) and the combination of elevated CO_2_ and elevated temperature (C+T+) in the T-FACE experiments of 2020 and 2021. Each data represents the mean value of three replications (with bars for ± standard errors of the mean). Different letters above the bars in the same time of each parameter indicate statistically significant differences (*p*< 0.05).

The vertical length of rice ShB lesions for YSBR1 (a resistant cultivar) was notably shorter than that for Lemont (a susceptible cultivar) under the four CO_2_ and temperature treatments in both 2020 and 2021 (**Figure 2**).

However, the effects of year, CO_2_, temperature and their combinations on vertical length of ShB lesions were similar between the two cultivars. The vertical length of ShB lesions measured on August 21, 23 and September 4 for both cultivars were significantly longer in 2021 than in 2020 (**Table 2** and **Figure 2**). Elevated CO_2_ had no significant effect on vertical length of ShB lesions for both cultivars at all measurement dates (**Table 2**, **Figure S2**). In contrast, elevated temperature significantly increased vertical length of ShB lesions for both cultivars at all measurement dates (**Table 2**; **Figure S2**). In addition, we observed significant interaction effects between year and CO_2_, between year and temperature on vertical length of ShB lesions for cv. Lemont on August 21 (**Table 2** and **Figures 2A, B**), and significant interaction effect among year, CO_2_ and temperature on vertical length of ShB lesions for cv. YSBR1 on August 23 and September 4 (**Table 2** and **Figures 2C, D**).

**Table 2:** Summary of analysis of variance (ANOVA) for the influence of year, CO_2_ and temperature on the dynamic change of vertical length of ShB lesions for cvs Lemont and YSBR1 in 2020 and 2021^a^.

Source of Variance	Vertical length of ShB lesions			
	Aug. 21	Aug. 23	Aug. 25	Sep. 4
Lemont				
Y	**<.001**	0.593	–	**<.001**
C	0.581	0.245	0.087	0.225
T	**<.001**	**<.001**	**<.001**	**<.001**
Y × C	**0.005**	0.106	–	0.097
Y × T	**0.003**	0.641	–	0.439
C × T	0.408	0.646	0.591	0.773
Y × C × T	0.068	0.218	–	0.114
YSBR1				
Y	**<.001**	**0.001**	–	**<.001**
C	0.267	0.105	0.278	0.057
T	**0.044**	**0.013**	**0.034**	**0.013**
Y × C	0.757	0.604	–	0.498
Y × T	0.075	**0.047**	–	0.141
C × T	0.439	0.495	0.164	0.644
Y × C × T	0.119	**0.039**	–	**0.020**

a Y, C and T stand for year, CO_2_ and temperature, respectively. Probability levels for significant differences are shown in the table, and P-value< 0.05 indicates a significant difference (in bold). “–” represents the data not existing.

### 3.2 Morphological parameters and their relationships to vertical length of ShB lesions

There were also significant differences between cv. Lemont and cv. YSBR1 in most morphological parameters of uninoculated plants under the four CO_2_ and temperature treatments (**Table 3**). Plant height (PH), flag leaf length (FLL), special leaf weight (SLW) and wax content of sheath (WCS) of cv. Lemont were significantly lower than those of cv. YSBR1 (**Table 3**). However, flag leaf width (FLW), stem wall thickness (SWT) and Si content of leaf (Si-L) of cv. Lemont were significantly higher than those of cv. YSBR1 (**Table 3**).

**Table 3:** Morphological parameters (SD between brackets) of uninoculated plants of cvs Lemont and YSBR1 at the booting stage under ambient condition (CT), elevated CO_2_ (C+T), elevated temperature (CT+), and the combination of elevated CO_2_ and elevated temperature (C+T+) in 2021^a^.

Varieties	Treatment	TN	PH (cm)	FLL (cm)	FLW (cm)	SLW (g m^-2^)	SSD (mm)	SWT (mm)	% WCS	Si-S (mg g^-1^)	Si-L (mg g^-1^)
**Lemont**	CT	8(1.00)	98.01(1.26)	37.47(1.54)	2.02(0.10)	61.55(0.87)	6.54(0.49)	1.77(0.11)	7.37(0.28)	0.24(0.02)	0.30(0.03)
	C+T	9(0.58)	100.97(2.91)	40.91(1.02)	2.05(0.13)	47.85(2.75)	6.57(0.21)	1.62(0.07)	7.85(0.14)	0.19(0.02)	0.29(0.01)
	CT+	8(0.58)	97.67(3.82)	38.91(0.70)	1.94(0.07)	54.96(0.99)	6.03(0.60)	1.67(0.09)	9.92(0.20)	0.26(0.01)	0.34(0.02)
	C+T+	9(0.58)	90.90(1.73)	34.01(1.58)	1.92(0.01)	53.95(1.82)	5.95(0.24)	1.83(0.12)	8.67(0.41)	0.19(0.01)	0.29(0.02)
**YSBR1**	CT	9(1.00)	117.96(1.26)	45.04(0.19)	1.59(0.08)	73.65(2.46)	5.89(0.40)	1.24(0.03)	10.41(0.16)	0.28(0.02)	0.26(0.02)
	C+T	9(0.58)	118.14(1.30)	44.99(0.56)	1.75(0.06)	64.12(1.39)	6.22(0.20)	1.17(0.04)	9.11(0.10)	0.20(0.02)	0.22(0.02)
	CT+	9(0.58)	112.30(0.78)	43.10(0.22)	1.58(0.08)	69.00(0.44)	6.34(0.27)	1.24(0.03)	12.13(0.16)	0.25(0.02)	0.26(0.02)
	C+T+	9(0.58)	111.00(1.43)	40.87(0.28)	1.60(0.09)	73.71(4.16)	6.39(0.20)	1.22(0.03)	9.51(0.35)	0.20(0.02)	0.24(0.03)
Probability of significance											
Lemont											
C		**0.021**	0.244	0.343	0.949	**<.001**	0.915	0.955	**0.040**	**<.001**	**0.041**
T		0.256	**0.009**	**0.006**	0.069	0.817	**0.047**	0.381	**<.001**	0.272	0.144
C × T		0.694	**0.013**	**<.001**	0.613	**<.001**	0.82	**0.031**	**0.001**	0.272	0.144
YSBR1											
C		0.694	0.449	**<.001**	0.088	0.138	0.28	**0.047**	**<.001**	**<.001**	**0.039**
T		0.694	**<.001**	**<.001**	0.111	0.129	0.091	0.311	**<.001**	0.289	0.598
C × T		0.694	0.323	**0.001**	0.139	**0.001**	0.419	0.187	**0.001**	0.289	0.598
V		0.011	**<.001**	**<.001**	**<.001**	**<.001**	0.697	**<.001**	**<.001**	0.128	**<.001**

a TN, triller number; PH, plant height; FLL, flag leaf length; FLW, flag leaf width; SLW, specific leaf weight; SSD, stem diameter; SWT, stem wall thickness; WCS, wax content of sheath; Si-S, Si content of stem; Si-L, Si content of leaf. V, C and T stand for variety, CO_2_ and temperature, respectively. Probability levels for significant differences are shown in table, and P-value< 0.05 indicates a significant difference (in bold).

Elevated CO_2_ significantly increased tiller number (TN) for cv. Lemont, but significantly decreased FLL, SWT, SLW, WCS, Si content of stem (Si-S) and Si-L for cv. Lemont and/or cv. YSBR1 (**Table 3**). Elevated temperature significantly increased WCS for both cultivars, but significantly decreased PH, FLL and stem diameter (SSD) for cv. Lemont and/or cv. YSBR1 (**Table 3**). In addition, there were significant interaction effects between CO_2_ and temperature on PH, FLL, SLW, SWT and WCS for cv. Lemont and/or cv. YSBR1 (**Table 3**).

We used Pearson correlation analysis to make clear which morphological parameters were correlated with vertical length of ShB lesions for cvs Lemont and YSBR1 under the four CO_2_ and temperature treatments. For cv. Lemont, vertical length of ShB lesions was significantly positive correlated with WCS and Si-L, but significantly negative correlated with FLW and SSD under the four CO_2_ and temperature treatments (**Figure S3A**). For cv. YSBR1, we also observed a significantly positive relationship between vertical length of ShB lesions and WCS under the four CO_2_ and temperature treatments (**Figure S3B**).

### 3.3 Physiological parameters and their relationships to vertical length of ShB lesions

There were also significant differences between cv. Lemont and cv. YSBR1 in most physiological parameters of inoculated and uninoculated plants under the four CO_2_ and temperature treatments (**Table 4**). Soluble sugar content of stems in cv. Lemont was significantly higher than that in cv. YSBR1 (**Table 4** and **Figure S4**). However, starch, cellulose, total phenol, flavonoid, lignin contents, MDA level, SOD, POD and PPO activities of stems in cv. Lemont were significantly lower than those in cv. YSBR1 (**Table 4** and **Figures S4–6**).

**Table 4:** Summary of analyses of variance (ANOVA) for the influence of inoculation, CO_2_ and temperature on physiological parameters in stems of cvs Lemont and YSBR1 in 2021^a^.

Source of Variance	Soluble sugar	Starch	Cellulose	Lignin	Total phenol	Flavonoid	MDA	SOD	POD	PPO
Lemont										
In	**<.001**	0.218	**<.001**	0.647	0.173	**<.001**	0.124	**<.001**	0.449	**<.001**
C	**<.001**	**<.001**	**<.001**	0.898	0.569	**<.001**	**<.001**	**<.001**	**0.001**	**<.001**
T	**<.001**	**0.619**	**0.012**	0.158	**0.018**	**0.001**	**<.001**	0.698	**0.001**	**<.001**
In × C	0.642	**<.001**	0.687	0.538	0.889	**<.001**	0.301	0.181	**0.020**	0.401
In × T	0.932	**<.001**	0.583	0.242	0.545	**<.001**	0.105	0.059	0.205	**<.001**
C × T	**0.011**	0.250	0.520	0.144	**<.001**	**<.001**	**<.001**	0.061	**0.005**	**<.001**
In × C × T	0.410	**0.002**	0.137	0.152	0.233	**<.001**	0.771	0.977	0.490	**<.001**
YSBR1										
In	**<.001**	**<.001**	**0.001**	**<.001**	**0.002**	**0.003**	**0.002**	0.063	**0.002**	**<.001**
C	0.237	**<.001**	**<.001**	**0.078**	**0.001**	0.202	**0.018**	**0.001**	**0.014**	**0.039**
T	**<.001**	**0.010**	**0.001**	0.454	**0.008**	**<.001**	**<.001**	**<.001**	**<.001**	0.899
In × C	0.865	**0.005**	0.065	**0.012**	0.640	**0.004**	0.228	0.883	**<.001**	**<.001**
In × T	**<.001**	**<.001**	**0.001**	**0.021**	0.764	0.707	0.573	0.088	**<.001**	**<.001**
C × T	0.273	0.974	**<.001**	0.057	0.264	**<.001**	**0.010**	0.113	0.314	**<.001**
In × C × T	0.116	**<.001**	**<.001**	**0.039**	**0.023**	**0.032**	0.440	0.405	0.148	**0.006**
V	**<.001**	**<.001**	**<.001**	**<.001**	**<.001**	**<.001**	**<.001**	**<.001**	**<.001**	**<.001**

a In, C, T and V stand for inoculation, CO_2_, temperature and variety, respectively. MDA, Malondialdehyde; SOD, superoxide dismutase; POD, peroxidase; PPO, polyphenol oxidase. Probability levels for significant differences are shown in the table, and P-value< 0.05 indicates a significant difference (in bold).

The effects of inoculation, CO_2_, temperature and their combinations on most physiological parameters were also significant for cvs Lemont and YSBR1 (**Table 4**). Inoculation of *R. solani* significantly increased total phenol and flavonoid contents, SOD activity and MDA level of stems for cv. Lemont and/or cv. YSBR1, but significantly decreased soluble sugar, starch, lignin and cellulose contents and POD activity of stems for cv. Lemont and/or cv. YSBR1 (**Table 4** and **Figures S4–6**). However, the effect of inoculation treatment on PPO activity of stems was opposite between the two cultivars (**Table 4** and **Figure S6**). Elevated CO_2_ significantly increased soluble sugar, starch and cellulose contents of stems for cv. Lemont and/or cv. YSBR1, but significantly decreased lignin and total phenol contents, and PPO activity of stems for cv. Lemont and/or cv. YSBR1 (**Table 4** and **Figures S4–6**). However, the effects of elevated CO_2_ on MDA level, SOD and POD activities of stems were opposite between the two cultivars (**Table 4** and **Figure S6**). Elevated temperature significantly increased soluble sugar and total phenol contents, and MDA level of stems for both cultivars, but significantly decreased starch, cellulose and flavonoid contents, and PPO activity of stems for cv. Lemont and/or YSBR1 (**Table 4** and **Figures S4–6**). However, the effect of elevated temperature on POD activity of stems differed between the two cultivars (**Table 4** and **Figure S6**). In addition, we observed significant interaction effects among inoculation, CO_2_ and temperature on starch, lignin, cellulose, total phenol and flavonoid contents, and PPO activity of stems for cv. Lemont and/or cv. YSBR1 (**Table 4**). There were significant interaction effects between inoculation and CO_2_ on starch, lignin and flavonoid contents, POD and PPO activities for cv. Lemont and/or cv. YSBR1 (**Table 4**). Significant interaction effects between inoculation and temperature on soluble sugar, starch, lignin, cellulose and flavonoid contents, and POD and PPO activities of stems were observed for cv. Lemont and/or cv. YSBR1 (**Table 4**). Significant interactions between CO_2_ and temperature for soluble sugar, cellulose, total phenol and flavonoid contents, MDA level, POD and PPO activities of stems were observed for cv. Lemont and/or cv. YSBR1 (**Table 4**).

**Figure 3:**
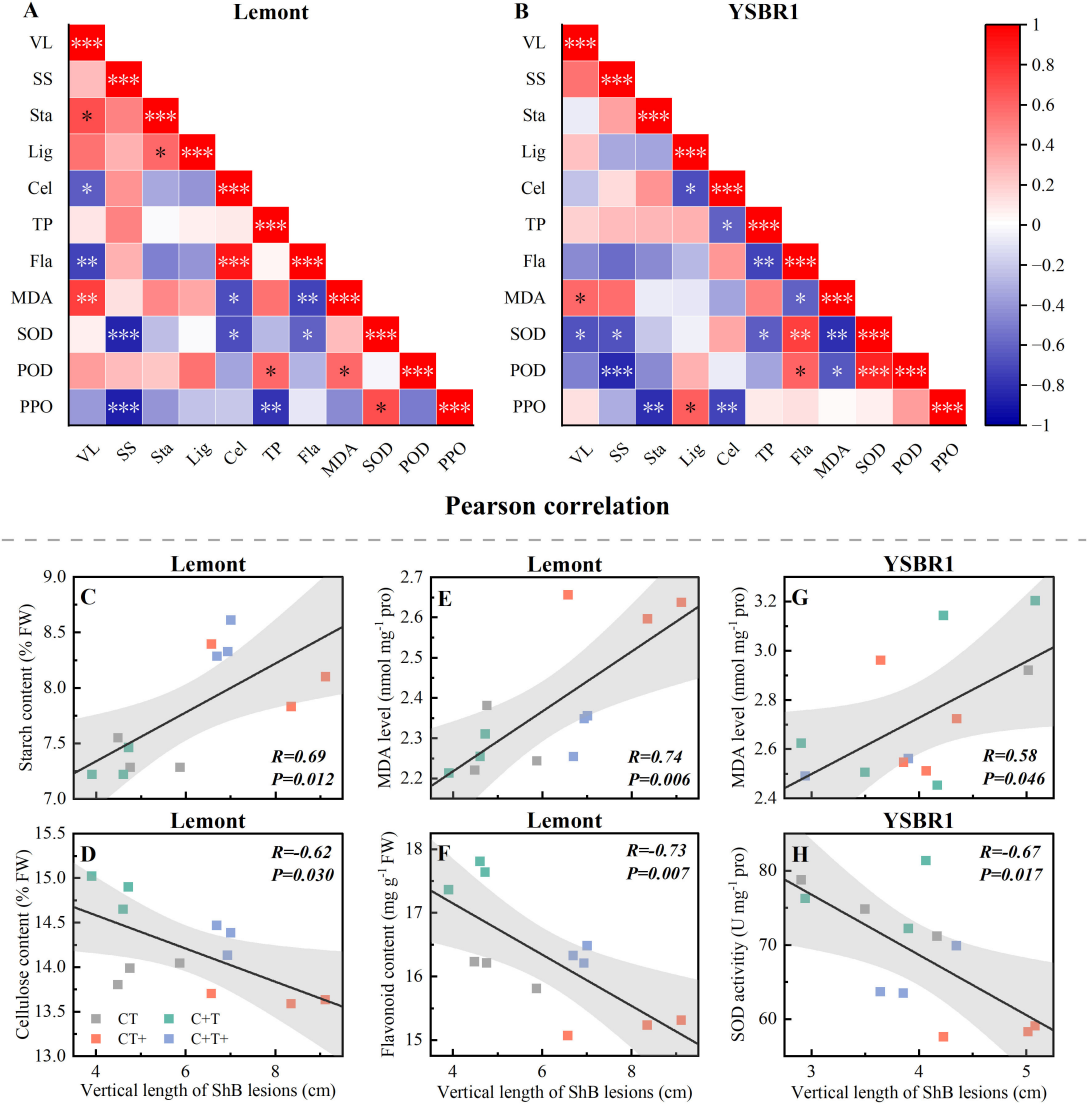
**(A, B)** Pearson correlation *R^2^* of vertical length of ShB lesions and physiological parameters in inoculated stems of cvs Lemont **(A)** and YSBR1 **(B)** under ambient condition (CT), elevated CO_2_ (C+T), elevated temperature (CT+), and the combination of elevated CO_2_ and elevated temperature (C+T+) in T-FACE experiments of 2021. VL, vertical length of ShB lesions; SS, soluble sugar; Sta, starch; Lig, lignin; Cel, cellulose; TP, total phenol; Fla, flavonoid; MDA, malondialdehyde; SOD, superoxide dismutase; POD, peroxidase; PPO, polyphenol oxidase. *p< = 0.05, **p< = 0.01, ***p< = 0.001. **(C-H)** Individual measures, regression line, correlation coefficient *R^2^*, confidence interval (95%), and *P*-value for cv. Lemont **(C)** Sta vs VL, **(D)** Cel vs VL, **(E)** MDA vs VL, **(F)** Fla vs VL, and cv. YSBR1 **(G)** MDA vs VL, **(H)** SOD vs VL.

Pearson correlation analysis showed that for cv. Lemont, the vertical length of ShB lesions was significantly positive correlated with starch content and MDA level of stems (**Figures 3A, C, E**), but significantly negative correlated with cellulose and flavonoid contents of stems (**Figures 3A, D, F**). For cv. YSBR1, the vertical length of ShB lesions was also significantly positive correlated with MDA level of stems (**Figures 3B, G**), but significantly negative correlated with SOD activity of stems (**Figures 3B, H**).

### 3.4 Above-ground biomass and grain yield

**Figure 4:**
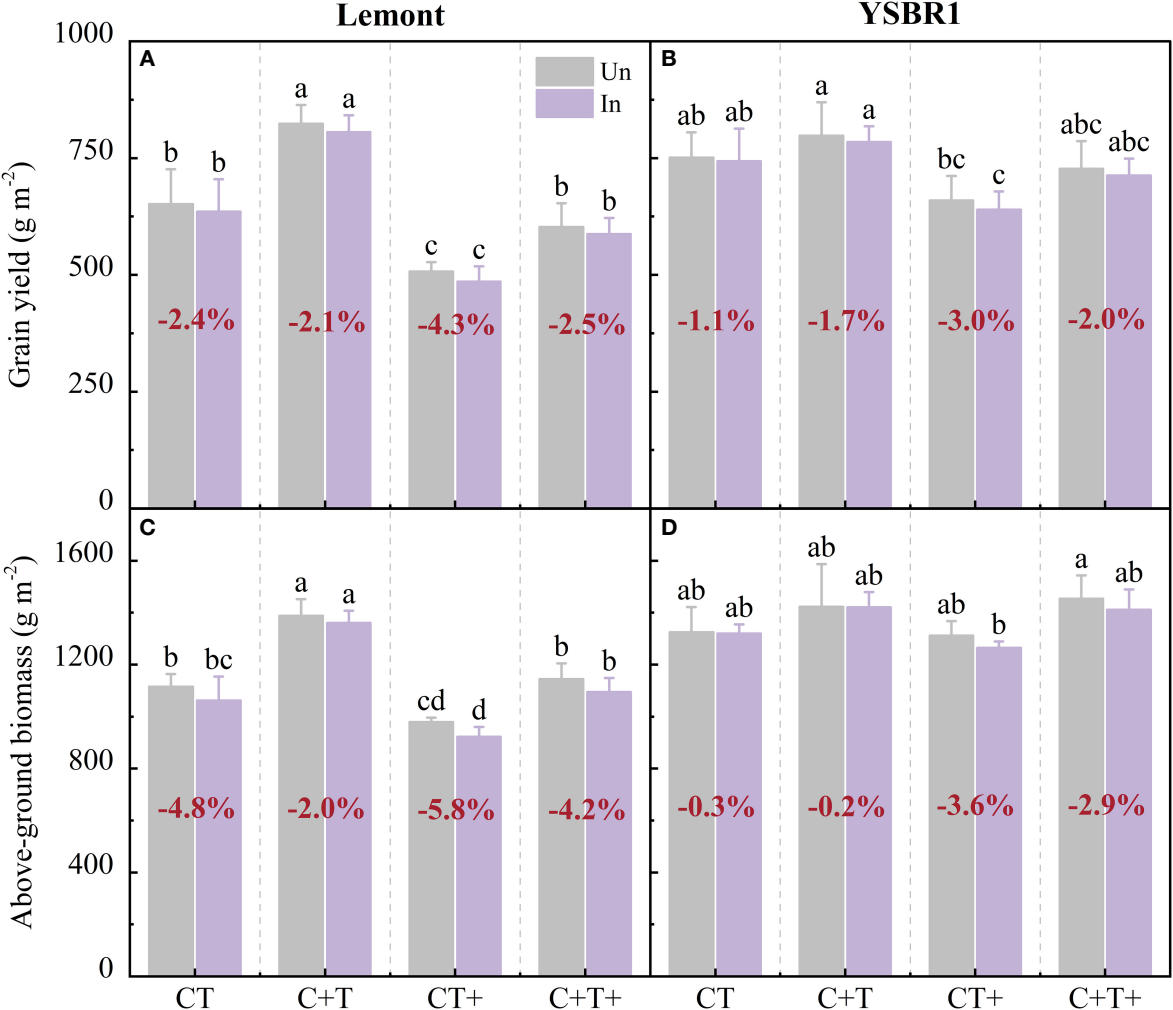
Grain yield and above-ground biomass at maturity for inoculation (In) and uninoculation (Un) of cvs Lemont **(A, C)** and YSBR1 **(B, D)** under ambient condition (CT), elevated CO_2_ (C+T), elevated temperature (CT+) and the combination of elevated CO_2_ and elevated temperature (C+T+) in the T-FACE experiments of 2021. Different letters above the bars in the same time of each parameter indicate statistically significant differences (*p*< 0.05). The bold number in red for each treatment in **(A, B)** is the average reduced ratio in grain yield of inoculated to uninoculated plants, and in **(C, D)** is the average reduced ratio in above-ground biomass of inoculated to uninoculated plants.

There was a significant interaction effect between CO_2_ and temperature on above-ground biomass for cvs Lemont and YSBR1 (**Table 5**). Elevated CO_2_ significantly increased grain yield and above-ground biomass for both cultivars (**Table 5**). However, elevated temperature significantly decreased grain yield for both cultivars (**Table 5** and **Figure 4**). Compared with ambient condition, the combination of elevated CO_2_ and elevated temperature decreased grain yield of uninoculated plants by 7.5% for cv. Lemont, and by 3.2% for cv. YSBR1. Thus, for uninoculated plants of the two cultivars, elevated CO_2_ could not compensate for the negative effect of elevated temperature on grain yield.

**Table 5:** Summary of analysis of variance (ANOVA) for the influence of inoculation, CO_2_ and temperature on above-ground biomass and grain yield of cvs Lemont and YSBR1 in 2021^a^.

Source of Variance	Lemont		YSBR1	
	Grain yield	Above-ground biomass	Grain yield	Above-ground biomass
In	0.388	0.062	0.538	0.500
C	**<.001**	**<.001**	**0.019**	**0.003**
T	**<.001**	**<.001**	**0.001**	0.742
In × C	0.952	0.716	0.996	0.964
In × T	0.958	0.801	0.883	0.563
C × T	0.085	**0.022**	0.555	0.541
In × C × T	0.909	0.857	0.909	0.979

a In, C and T stand for inoculation, CO_2_ and temperature, respectively. Probability levels for significant differences are shown in the table, and P-value< 0.05 indicates a significant difference (in bold).

Inoculated plants with *R. solani* further decreased above-ground biomass and grain yield of both cultivars under elevated temperature and the combination of elevated CO_2_ and elevated temperature. Compared with uninoculated plants, inoculated plants with *R. solani* decreased above-ground biomass and grain yield by 5.8–4.3% for cv. Lemont and by 3.6–3.0% for cv. YSBR1 under elevated temperature, and by 4.2–2.5% for cv. Lemont and by 2.9–2.0% for cv. YSBR1 under the combination of elevated CO_2_ and elevated temperature (**Figure 4**).

## 4 Discussion

### 4.1 Elevated CO_2_ and temperature affect the risk of ShB severity

Previous studies found that indica cultivars exhibited higher level of resistance to ShB than japonica cultivars (Zuo et al., 2008; Sharma et al., 2009; Jia et al., 2012; Molla et al., 2020). This was consistent with our results that for inoculated plants with *R. solani*, the vertical length of ShB lesions in cv. YSBR1 (indica) was shorter than that in cv. Lemont (japonica) under four CO_2_ and temperature treatments in 2020 and 2021 (**Figure 2** and **Figure S2**). Thus, growing indica subspecies with high resistance to ShB may reduce the severity of ShB under future climate change. In addition, we found that there was a significant difference between years in the vertical length of ShB disease in both cultivars under four CO_2_ and temperature treatments (**Figure 2** and **Table 2**). The vertical length of ShB lesions was much longer in both cultivars in 2021 under four CO_2_ and temperature treatments than in 2020 (**Figure 2** and **Table 2**).

Elevated CO_2_ has been reported to increase the horizontal spread of ShB, but not upward spread of the ShB lesions (Kobayashi et al., 2006). Eastburn et al. (2011) suggested that elevated CO_2_ increases leaf number, leaf area, canopy size and density, and alters canopy temperature and microclimate humidity, which may promote fungal diseases. However, our results showed that elevated CO_2_ had no significant effect on the vertical length of ShB disease in both cvs YSBR1 and Lemont in 2020 and 2021 (**Figure 2** and **Table 2**). In contrast, elevated temperature significantly increased the vertical length of ShB lesion in both cultivars at all measurement dates in both years (**Figure 2** and **Table 2**). Previous studies showed that the increased air temperatures had a significant effect on plant diseases (Chakraborty et al., 2000; Chakraborty, 2001). Higher temperatures were positively correlated with an overall increase in spot blotch severity, particularly nighttime temperatures (Sharma et al., 2007). Our results showed that 19 days after inoculation (September 4) in both years, C+T+ increased vertical length of lesions by 20.5–38.4% for cv. Lemont and by -0.5–5.8% for cv. YSBR1, compared with CT. Thus, the combination of elevated CO_2_ and elevated temperature under future climate change largely increase the risk of rice ShB, especially for ShB susceptible rice cultivars.

### 4.2 Exploring factors that affect ShB severity under CO_2_ and temperature treatments

Among meteorological factors, high air temperature combined with high relative humidity in the forenoon is one of major factors to predispose ShB development in rice (Castilla et al., 1996; Biswas et al., 2011). Maximum dispersal of ShB was observed in a temperature range of 25–30°C and relative humidity of 80–100%, and a maximum temperature around 34°C and a minimum temperature around 26°C were found to be favorable for ShB dispersal after its establishment in the field (Thind et al., 2008; Biswas et al., 2011; Bhukal et al., 2015; Senapati et al., 2022). In our study, the average daily air temperature after inoculation in 2021 was 0.8°C lower than in 2020, while the average relative humidity after inoculation in 2021 was 9% higher than in 2020 (**Figure S1**). The higher relative humidity after inoculation may be related to the greater vertical length of ShB lesions in 2021 than in 2020 for cvs Lemont and YSBR1 under four CO_2_ and temperature treatments (**Figure S1** and **Table 2**).

Many studies have investigated which morphological and physiological parameters were related to susceptibility to ShB in rice under ambient conditions, but with inconsistent results (Zou et al., 2000; Pinson et al., 2005; Ma et al., 2006; Willocquet et al., 2012; Zuo et al., 2014b; Dey et al., 2016; Sathe et al., 2021; Senapati et al., 2022). The wax layer is the first barrier for plants, and has the function of resisting pathogen invasion in the natural environment (Jetter and Schaffer, 2001; Chen et al., 2003). Disease resistance is positively correlated with stem thickness under artificial inoculation of *R. solani* (Dey et al., 2016). Some studies of artificial inoculation showed higher Si content in rice suppressed lesion development, resulting in increased resistance to *R. solani* (Rodrigues et al., 2003a; Rodrigues et al., 2003b; Schurt et al., 2014; Sathe et al., 2021). Rice plants respond to *R. solani* infecting, by activating various signaling pathways and producing antimicrobial compounds to prevent it from colonizing (Senapati et al., 2022). Some studies suggest that carbohydrate metabolism is the major area of metabolic changes when rice responses to the *R. solani* attack (Karmakar et al., 2019; Molla et al., 2020). Flavonoids produced from phenolic metabolism provide resistance to pathogens (Ertani et al., 2011) and SOD activity, as a key enzyme, provides initial resistance information (Zhang et al., 2006). Our results found that plant height, flag leaf length, specific leaf weight and wax content of sheath were significantly higher in cv. YSBR1 than in cv. Lemont under four CO_2_ and temperature treatments (**Table 3**). In addition, the starch, cellulose, total phenol, flavonoid and lignin contents, SOD, POD and PPO activities of stems in cv. YSBR1 were significantly higher than those in cv. Lemont under all treatments (**Table 4** and **Figures S4–6**). These results can be used to explain that the vertical length of ShB lesions in cv. YSBR1 (indica) was shorter than that in cv. Lemont (japonica) under four CO_2_ and temperature treatments in 2020 and 2021 (**Figure 2**). Zuo et al. (2014b) also found that overall antimicrobial compounds were higher in YSBR1 under ambient conditions than Lemont.

Pearson correlation analysis showed that the vertical length of ShB lesions was significantly positively correlated with wax content of sheath in both cultivars and with Si content of stems in cv. Lemont, but significantly negatively correlated with flag leaf width or stem diameter in cv. Lemont under four CO_2_ and temperature treatments (**Figure S3**). In addition, the vertical length of ShB lesions was significantly positively correlated with MDA level of inoculated stems in both cultivars under four CO_2_ and temperature treatments (**Figure 3**). Our results showed that elevated temperature (CT+ and C+T+) significantly increased MDA level of stems in both cultivars (**Table 4** and **Figure 3**), consistent with Bamagoos et al. (2021). Inoculation treatments significantly increased MDA level of stems in cv. YSBR1. Overall, higher MDA level of stems may be related to longer vertical length of ShB for inoculated plants of both cultivars under elevated temperature (CT+ and C+T+).

### 4.3 The losses of biomass and yield were correlated with the severity of ShB

The saprophytic nature of *R. solani* and the broad host range of pathogen have led to the spread and persistence of the fungus in all rice-producing areas (Lee and Rush, 1983). Reportedly, the pathogen of *R. solani* has spread in almost all rice growing areas in the world and economic losses of up to 58% in rice yield have been revealed (Chahal et al., 2003; Molla et al., 2020). Inoculation significantly increased the sheath blight severity and incidence, and caused yield losses ranging from 4% in moderately susceptible to 21% in highly susceptible rice plants (Groth and Bond, 2007). Wu et al. (2013) found that artificial inoculation at panicle initiation stage done to achieve uniform disease development would significantly increase ShB severity and cause yield reduction from 12.1% to 48.6% in all experimental seasons. Meanwhile, Wu et al. (2013) also concluded that sheath blight infestation reduced rice yield mainly by decreasing biomass production. Our results showed that for uninoculation of plants, the combination of elevated CO_2_ and elevated temperature decreased grain yield by 3.2–7.5% for cvs Lemont and YSBR1. Inoculation with *R. solani* further decreased grain yield by 3.0–4.3% under elevated temperature, and by 2.0–2.5% under the combination of elevated CO_2_ and elevated temperature (**Figure 4**). Thus, elevated CO_2_ could not offset the negative effects of elevated temperature on rice yield under future climate change. The combination of elevated CO_2_ and elevated temperature further reduced rice yield by increasing ShB severity.

Our previous study showed that improving nitrogen uptake before heading and selecting heat-tolerant varieties are crucial in minimizing rice yield loss caused by the combination of elevated CO_2_ and elevated temperature under future climate change (Cai et al., 2016). However, Savary et al. (1995) found that the use of high doses of nitrogen fertilizer and the adoption of semi-dwarf high-yielding varieties led to a sharp increase in the incidence of sheath blight disease. In double-rice cultivation, short-stature and early-maturing varieties were usually used, the incidence of sheath blight was usually higher than in single-rice cultivation (Sharma et al., 1995; Wang, 2006). In the absence of effective host plant resistance against *R. solani*, the management of sheath blight disease is largely managed through the use of chemicals, such as fungicides (Naik et al., 2017). Meanwhile, the use of a single chemical with the same application over a long period of time leads to the development of resistance in the fungus (Uppala and Zhou, 2018). The chemical control method has an advantage of reducing disease incidence, spread and increasing yield, but it also has several disadvantages, such as environmental hazards (Senapati et al., 2022). Therefore, reasonable agronomic management practices are required to improve both ShB disease resistance and grain yield of rice under future climate change.

## 5 Conclusion

This study was first to explore the effects of elevated CO_2_ and temperature on the severity of rice ShB using the T-FACE system. Our results showed that elevated temperature, but not elevated CO_2_, significantly increased the vertical length of rice ShB lesions for both susceptible and resistant cultivars (cvs Lemont and YSBR1). The risk of rice ShB severity was increased under the combination of elevated CO_2_ and elevated temperature for both cultivars. A significant increase in MDA level of stems may be related to a significant increase in the vertical length of ShB lesions under the combination of elevated CO_2_ and elevated temperature. Elevated CO_2_ could not offset the negative effect of elevated temperature on rice yields under future climate change. The combination of elevated CO_2_ and elevated temperature further reduced rice yields by increasing ShB severity. High resistant variety and reasonable agronomic managements are needed to decrease the risk of ShB disease and increase grain yield of rice under future climate change.

## Data availability statement

The raw data supporting the conclusions of this article will be made available by the authors, without undue reservation.

## Author contributions

MS and CZ conceived and designed the experiment; MS, JQ, CM, DW, LS, WW, XG, XY, YT, JZ and GL performed the experiment; MS and CC analyzed data and wrote the manuscript; CC and LS revised the manuscript. All authors contributed to the article and approved the submitted version.
