# Supplementary material for: Elevated CO2 and temperature under future climate change increase severity of rice sheath blight
Source: Front Plant Sci. 2023 Jan 26;14:1115614. doi: 10.3389/fpls.2023.1115614 (PMC9909553; doi:10.3389/fpls.2023.1115614)
Supplement: Supplementary file 1 [file DataSheet_1.docx]

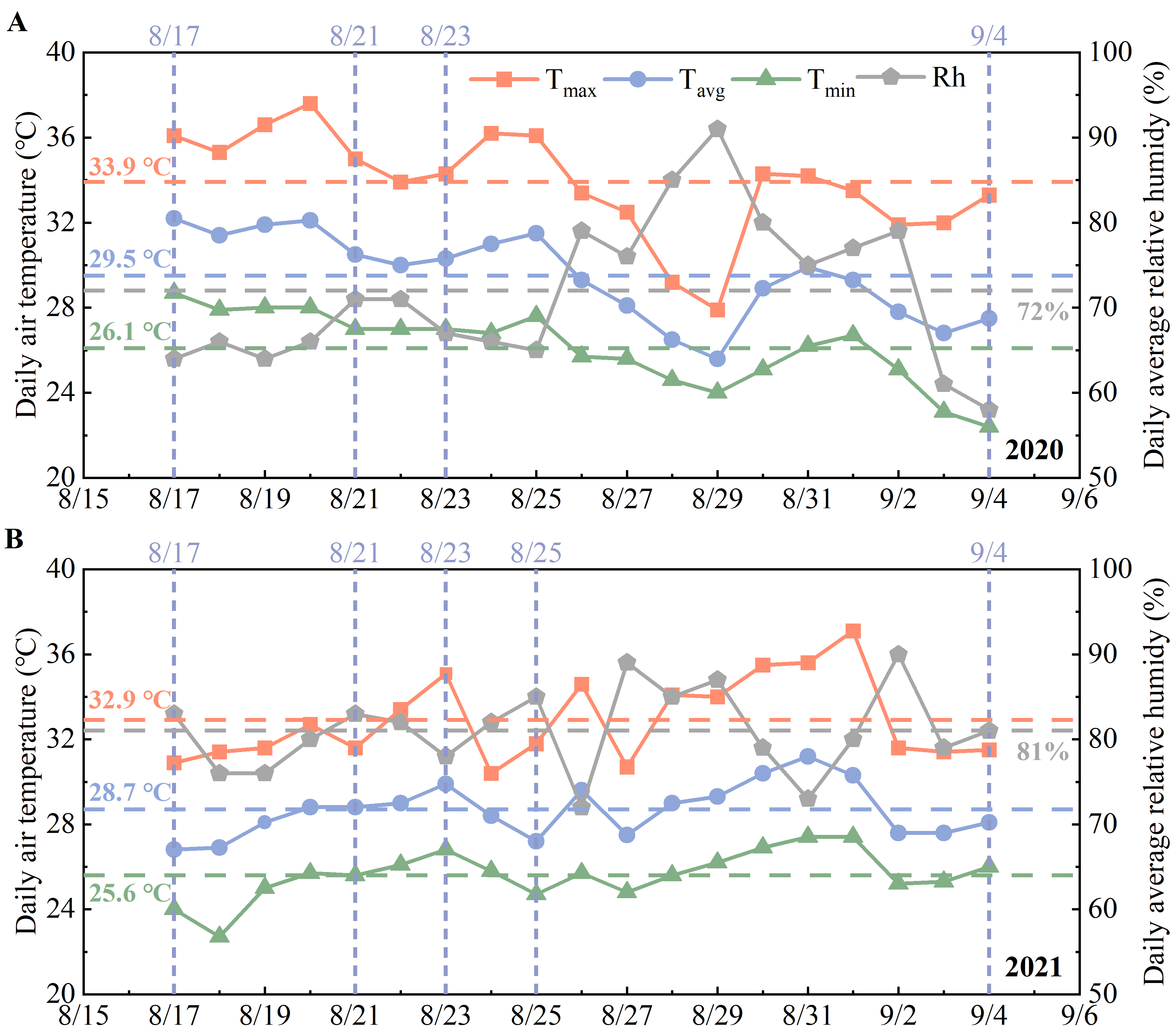


**Figure S1** Daily maximum (*T_max_*), average (*T_avg_*) and minimum (*T_min_*) air temperature, and daily average relative humidity (RH) from Aug. 17 to Sep. 4, 2020 (A) and 2021 (B), respectively. The dotted lines in horizontal direction with marked number represent the mean of *T_max_* (in orange), *T_avg_* (in blue), *T_min_* (in green) and RH (in grey) during all inoculation period. The dotted lines in the vertical direction stand for the dates of inoculated day (Aug. 17) and lesion length surveys in 2020 and 2021, respectively.


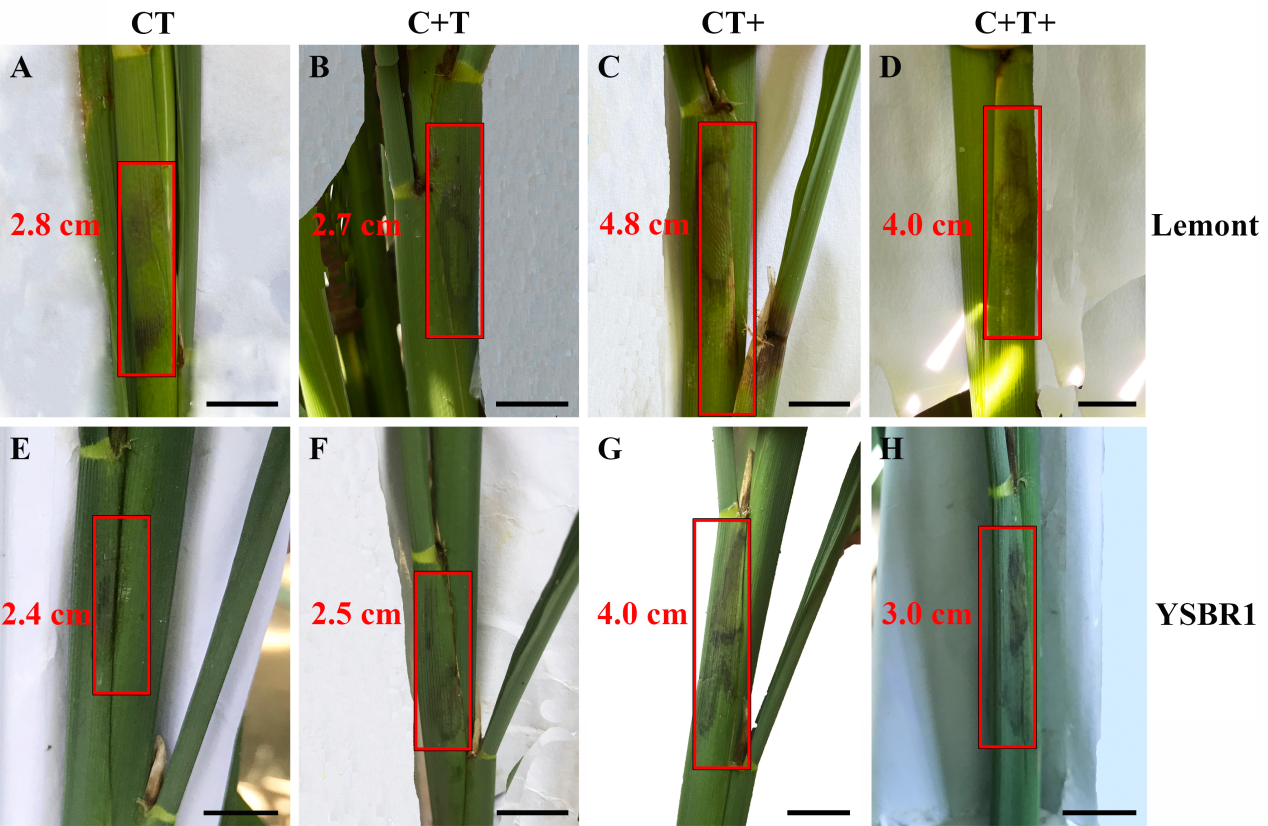


**Figure S2** Phenotypic photos of rice ShB lesions for cvs Lemont (A-D) and YSBR1 (E-H) on August 21, 2021 under the conditions of ambient CT (A, E), elevated CO_2_ C+T (B, F), elevated temperature CT+ (C, G) and the combination of elevated CO_2_ and elevated temperature C+T+ (D, H). The rectangle box and bold number in red in each panel are ShB lesions and vertical length of ShB lesions, respectively. Scale bars in (a–h) = 1 cm.


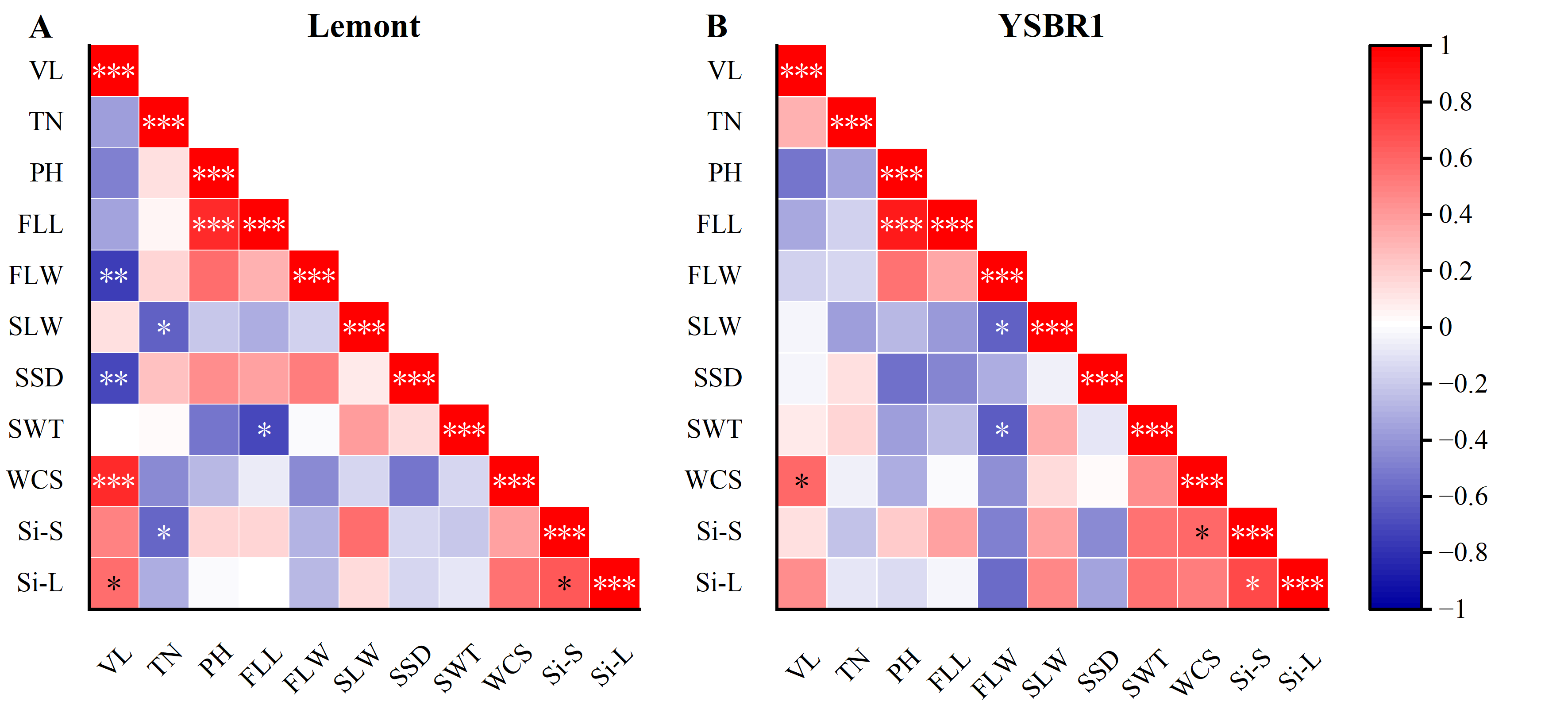


**Figure S3** Pearson correlation *R^2^* of vertical length of ShB lesions on Aug. 25, 2021 and morphological parameters of uninoculated plants on Aug. 26, 2021 for cvs Lemont (A) and YSBR1 (B). Negative correlations (red) and positive (blue). VL, vertical length of ShB lesions; TN, triller number; PH, plant height; FLL, flag leaf length; FLW, flag leaf width; SLW, specific leaf weight; SSD, stem diameter; SWT, stem wall thickness; WCS, wax content of sheath; Si-S, Si content of stem; Si-L, Si content of leaf. * *p* < = 0.05, ** *p* < = 0.01, *** *p* < = 0.001.


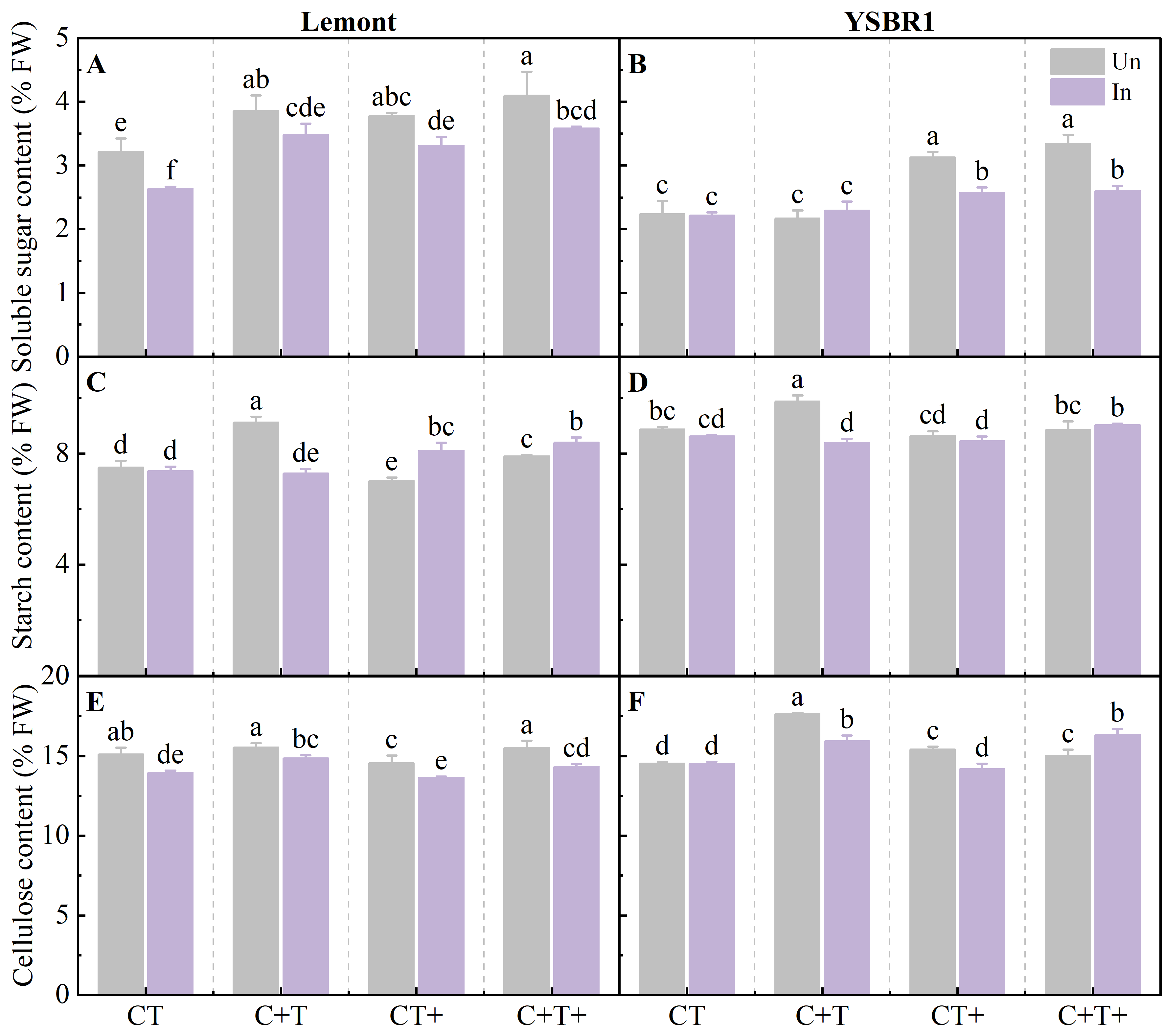


**Figure S4** Carbohydrate metabolites including soluble sugar, starch and cellulose contents of stems for inoculation (In) and uninoculation (Un) of cvs Lemont (A, C, E) and YSBR1 (B, D, F) under ambient condition (CT), elevated CO_2_ (C+T), elevated temperature (CT+) and the combination of elevated CO_2_ and elevated temperature (C+T+) on Aug. 26, 2021. Each data represents the mean value of three replications (with bars for ±standard errors of the mean). Different letters above the bars in the same time of each parameter indicate statistically significant differences (*p* < 0.05).


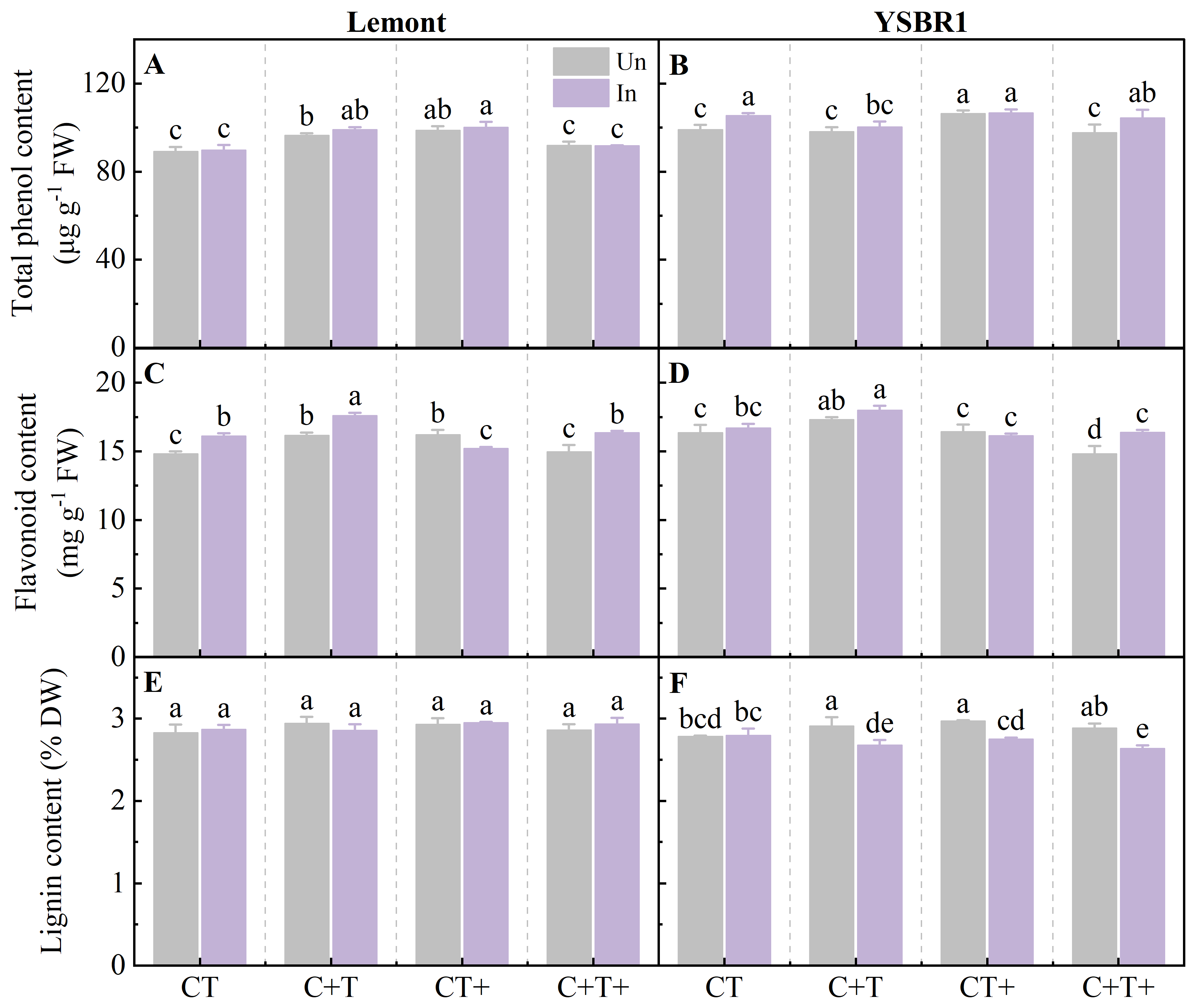


**Figure S5** Phenolic metabolites including total phenol, flavonoid and lignin contents of stems for inoculation (In) and uninoculation (Un) of cvs Lemont (A, C, E) and YSBR1 (B, D, F) under ambient condition (CT), elevated CO_2_ (C+T), elevated temperature (CT+) and the combination of elevated CO_2_ and elevated temperature (C+T+) on Aug. 26, 2021. Each data represents the mean value of three replications (with bars for ±standard errors of the mean). Different letters above the bars in the same time of each parameter indicate statistically significant differences (*p* < 0.05).


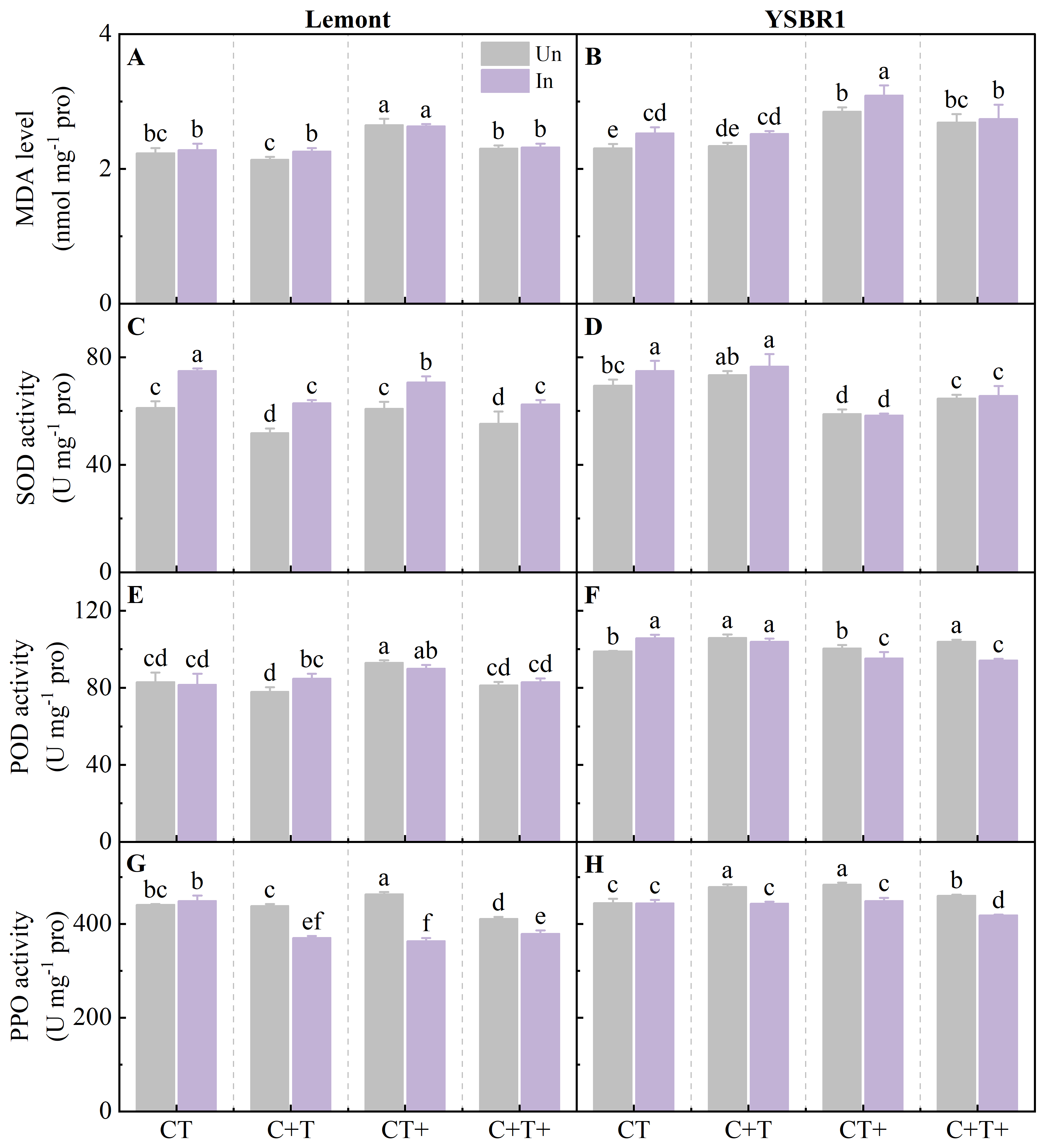


**Figure S6** Membrane lipid peroxidation (MDA level) and antioxidant enzymes including SOD, POD and PPO activity of stems for inoculation (In) and uninoculation (Un) of cvs Lemont (A, C, E, G) and YSBR1 (B, D, F, H) under ambient condition (CT), elevated CO_2_ (C+T), elevated temperature (CT+) and the combination of elevated CO_2_ and elevated temperature (C+T+) on Aug. 26, 2021. Each data represents the mean value of three replications (with bars for ±standard errors of the mean). Different letters above the bars in the same time of each parameter indicate statistically significant differences (*p* < 0.05).
